# Effective Photodynamic Therapy with Ir(III) for Virulent Clinical Isolates of Extended-Spectrum Beta-Lactamase *Klebsiella pneumoniae*

**DOI:** 10.3390/pharmaceutics13050603

**Published:** 2021-04-22

**Authors:** Constanza Núñez, Annegrett Palavecino, Iván A. González, Paulina Dreyse, Christian Erick Palavecino

**Affiliations:** 1 Laboratorio de Microbiología Celular, Instituto de Investigación y Postgrado, Facultad de Ciencias de la Salud, Universidad Central de Chile, Santiago 8330546, Chile; constanza.nunezc@alumnos.ucentral.cl (C.N.); Annegrett.palavecino@alumnos.ucentral.cl (A.P.); 2 Laboratorio de Química Aplicada, Instituto de Investigación y Postgrado, Facultad de Ciencias de la Salud, Universidad Central de Chile, Santiago 8330546, Chile; ivan.gonzalez@ucentral.cl; 3 Departamento de Química, Universidad Técnica Federico Santa María, Av. España 1680, Casilla 2390123, Valparaíso 2390123, Chile; paulina.dreyse@usm.cl

**Keywords:** antibiotic resistance, virulence factors, *Klebsiella pneumoniae*, photodynamic therapy

## Abstract

Background: The extended-spectrum beta-lactamase (ESBL) *Klebsiella pneumoniae* is one of the leading causes of health-associated infections (HAIs), whose antibiotic treatments have been severely reduced. Moreover, HAI bacteria may harbor pathogenic factors such as siderophores, enzymes, or capsules, which increase the virulence of these strains. Thus, new therapies, such as antimicrobial photodynamic inactivation (aPDI), are needed. Method: A collection of 118 clinical isolates of *K. pneumoniae* was characterized by susceptibility and virulence through the determination of the minimum inhibitory concentration (MIC) of amikacin (Amk), cefotaxime (Cfx), ceftazidime (Cfz), imipenem (Imp), meropenem (Mer), and piperacillin–tazobactam (Pip–Taz); and, by PCR, the frequency of the virulence genes K2, magA, rmpA, entB, ybtS, and allS. Susceptibility to innate immunity, such as human serum, macrophages, and polymorphonuclear cells, was tested. All the strains were tested for sensitivity to the photosensitizer PSIR-3 (4 µg/mL) in a 17 µW/cm^2^ for 30 min aPDI. Results: A significantly higher frequency of virulence genes in ESBL than non-ESBL bacteria was observed. The isolates of the genotype K2+, ybtS+, and allS+ display enhanced virulence, since they showed higher resistance to human serum, as well as to phagocytosis. All strains are susceptible to the aPDI with PSIR-3 decreasing viability in 3log10. The combined treatment with Cfx improved the aPDI to 6log10 for the ESBL strains. The combined treatment is synergistic, as it showed a fractional inhibitory concentration (FIC) index value of 0.15. Conclusions: The aPDI effectively inhibits clinical isolates of *K. pneumoniae*, including the riskier strains of ESBL-producing bacteria and the K2+, ybtS+, and allS+ genotype. The aPDI with PSIR-3 is synergistic with Cfx.

## 1. Introduction

*Klebsiella pneumoniae* is one of the major health-associated infection (HAIs) producers worldwide, including pneumonia, urinary tract, and bloodstream infections [1,2]. Moreover, HAI-producing *K. pneumoniae* strains progressively accumulate more multiple-drugs resistance (MDR), including extended-spectrum β-lactamases (ESBL) and carbapenemases, such as KPC [3,4,5]. Treatment options for infections caused by MDR strains of *K. pneumoniae* are severely reduced to colistin and tigecycline [6,7,8]. The high MDR shown by strains of *K. pneumoniae* does not fully explain its notable success as one of the most important agents of HAIs, suggesting the participation of other factors [9]. An increasing number of authors suggest that increased bacterial survival during infections is related to virulence factors [1,3,9,10,11,12,13,14].

Virulence factors shown by *Klebsiella pneumoniae* strains are common to enterobacteria, such as siderophores, enzymes, and capsules [9]. These factors may be part of genes conserved amongst all *K. pneumoniae*, called the core set of genes, but other genes vary in frequency between strains, and they are part of the accessory set of genes [9]. During colonization and infection, *K. pneumoniae* requires the expression of several core and accessory genes to deal with, for example, the nutritional stress due to nutrient sequestration or the immune response of the host [15]. For instance, during lung infection, the acquisition of iron is facilitated by the siderophores enterobactin (*ent*B) and yersiniabactin (*ybt*S) genes encoded on the bacterial chromosome [1,16]. Like iron, during infections of the lungs, urinary tract, and bloodstream, access to nitrogen is limited. The *all*S gene allows *K. pneumoniae* to use allantoin degradation as an alternative nitrogen source [17,18]. The polysaccharide capsule is involved, among others, in resistance to death by complement-mediated opsonophagocytosis [9], and inhibition of macrophages [19,20]. Some capsular varieties (such as K2) may produce hypervirulent strains with characteristic phenotypes such as the hypermucoviscous [19]. Some hypervirulent phenotypes are associated with the expression of the mucoviscosity-associated gene A (*mag*A) and the regulator of the mucoid phenotype A gene (*rmp*A) [21]. Moreover, the *rmp*A gene increases the ability of ESBL-producing strains to resist the bactericidal activity of serum and phagocytosis [22]. Since the virulence factors increase bacterial survival and may influence antibiotic resistance expression, the composition of the pool of the virulence gene of *K. pneumoniae* strains may be associated with the antibiotic susceptibility pattern [9,13].

Due to the emergence of multi-drug resistance (MDR), the deficit of new antibiotics is one of the most pressing threats to human health in the 21st century [23]. Given the high risk to public health caused by the deficiency of new antibiotics, the use of complementary antimicrobial therapies non-antibiotic-based, such as antibacterial photodynamic inactivation (aPDI), emerges as a promising alternative [24]. The aPDI may support the lack of antibiotic therapies against MDR and KPC strains [24,25]. The aPDI is a procedure based on the use of photosensitizer (PS) compounds to produce light-activated local cytotoxicity (photooxidative stress) [26]. The PSs work by energy absorbs of a specific wavelength in the UV–Vis range, which is then transferred to molecular oxygen in solution to produce reactive oxygen species (ROS) [27]. Molecular O_2_ can accept this energy together with electrons, or it can only undergo a one-electron reduction to produce superoxide anion radical (O_2_^•−^), hydrogen peroxide (H_2_O_2_), and hydroxyl radical (HO^•^) [28,29]. The energy transferred to the O_2_ without one-electron reduction produces singlet oxygen (^1^O_2_) [27]. The ROS production generates photooxidative stress induced by aPDI, which occurs mainly due to the action of ^1^O_2_. The oxidative action of ^1^O_2_ occurs on organic molecules close to the PS through concerted addition reactions of alkene groups [30]. These organic molecules can be structural proteins or lipids of the bacterial envelope; therefore, the damage occurs to the cell wall plasma membrane or other bacterial structures, leading to nonspecific cell death [27,31]. Previously, a PS compound based on a polypyridine Ir(III) complex (PSIR-3, see Figure 4) demonstrated aPDI activity, inhibiting the bacterial growth of CKP and synergism with imipenem [32,33]. In this work, we determine the frequency of the K2, *ent*B, *yb*tS, *all*S, *rmp*A, and *mag*A virulence genes, in a population of 118 clinical isolates of *K. pneumoniae* and evaluate their association to the susceptibility pattern to several antibiotics. Our data show that virulence factors K2^+^, *ybt*S^+^, and *all*S^+^ were associated with a modification in the bacterial minimum inhibitory concentrations (MICs) and increased ESBL production probability. These virulence genes significantly increased the survival of the bacteria, to innate immunity. These more virulent ESBL bacteria were all susceptible to the aPDI treatment with PSIR-3 and demonstrate synergism with cephotaxime.

## 2. Materials and Methods

### 2.1. Bacterial Isolates

The ethics committee of the Faculty of Health Sciences, Central University of Chile, and the Central Metropolitan Health Service of Chile (MHSC), Act number: N 124/07, approved the study protocol and the informed consent form. The clinical isolates of *K. pneumoniae* were obtained from 122 samples from unrelated patients, received in the bacteriology laboratory of “Hospital el Carmen” (HEC) for a period of six months (2017/2018). The HEC is a complex hospital with 412 beds and serving a population of more than 600,000 inhabitants. All clinical isolates were identified as *K. pneumoniae* following the protocols of the Institute of Clinical and Laboratory Standards (CLSI) [34]. As controls, the virulent strain (ATCC 43816 KPPR1) of susceptible *K. pneumoniae* (K2^+^, *ybt*S^+^, and *all*S^+^) [35] and the MDR KPC^+^ ST258 (KP35) (K2^−^, *ybt*S^−^ and *all*S^−^) [36] strains were also included.

### 2.2. Antimicrobial Susceptibility Testing

The MIC of the antimicrobial agents were determined in 96-well plates by micro-dilution methodology in cations-adjusted Mueller-Hinton (ca-MHB) broth. Inoculum of 1 × 10^6^ colony forming units (CFUs)/mL of each clinical isolate was mixed with decreasing concentrations of each antibiotic and incubated overnight at 37 °C following the CLSI recommendations. The MIC for each antibiotic was determined as the last dilution in which no bacterial growth occurred, and the susceptibility intervals were assigned based on the cutoff points established by the CLSI (2018) for amikacin (Amk), cefotaxime (Cfx), ceftazidime (Cfz), imipenem (Imp), meropenem (Mer), and piperacillin–tazobactam (Pip–Taz). According to the CLSI protocols [34], clinical isolates were strains considered ESBL-producing when resistant to cefotaxime but susceptible to the combination of Cfx/clavulanic acid. Values are presented as the median in mg/L and interquartile range (IQR).

### 2.3. DNA Extraction and PCR Amplification

The total DNA was obtained from stationary bacterial cultures in LB broth using the phenol-chloroform methodology. In brief, pelleted bacteria were suspended in 300 µL of PBS and mixed with 300 µL of phenol: chloroform (25:24 vol:vol); the final mix was stirred well in a vortex and centrifuged at 13,000× *g* at 4 °C for 15 min. The aqueous phase was mixed 1:1 with chloroform and centrifuged as above. Genomic and plasmidial DNA contained in the aqueous phase was precipitated in 0.6 vol of 2-propanol and sedimented at 13,000× *g* at 4 °C for 20 min. The nucleic acids were washed twice with 70% ethanol and resuspended in Tris-EDTA buffer (10 mM Tris-HCl, 1 mM disodium EDTA, pH 8.0). PCR reactions were performed, using 0.5 µM of each specific primer pair listed in Table 1, in 1× master mix GoTaq (Promega). The amplification was carried out at a final volume of 20 µL in a Veriti (Applied Biosystem) PCR machine with an initial denaturation step of 10 s at 95 °C, followed by 35 cycles of 10 s at 95 °C, 15 s at 58 °C, and 30 s of extension at 72 °C. A final extension step of 7 min at 72 °C was included, and PCR products were visualized on a 1.7% agarose gel.

### 2.4. K. Pneumoniae Survival to Innate Immunity

To evaluate the survival of *K. pneumoniae* to the innate immunity, the bactericidal activity of normal human serum (NHS), as well as phagocytosis by human macrophages (MΦ), and polymorphonuclear (PMN) cells was determined. Both serum and leukocytes were obtained from blood samples of healthy voluntary donors who had not taken any antibiotic or anti-inflammatory medication for at least ten days before the day of sampling.

#### 2.4.1. Susceptibility to Normal Human Serum

Serum susceptibility was carried out as before [37]; in brief, 75 µL of pooled NHS were mixed with 25 µL suspension containing 2 × 10^6^ CFUs of each isolate in a 96-well plate. As a control, bacterial cultures of each isolate were mixed with PBS. The mixtures were incubated at 37 °C for 3 h, and viable bacteria were enumerated by serial micro-dilution and colony counting on ca-MH agar plates. Serum resistance is expressed as viable bacteria in CFUs/mL compared to untreated isolates controls.

#### 2.4.2. Susceptibility to Phagocytosis by Macrophages and Polymorphonuclear Cells

The separation of mononuclear and polymorphonuclear cells was performed by centrifugation in a gradient density column of Histopaque (Sigma-Aldrich, St. Louis, MO, USA), following the manufacturer instructions. Monocytes were selected from other mononuclear cells by incubation in RPMI-1640 medium (Sigma-Aldrich) without fetal bovine serum (FBS) and differentiated into macrophages incubating during 7–9 days in RPMI-1640 10% FBS at 37 °C with 5% CO_2_ [38]. The macrophage monolayer was infected with 2.5 × 10^7^ CFUs with a multiplicity of infection (MOI) of 50:1 of each bacterial isolate and centrifuged at 200× *g* for 5 min, to synchronize phagocytosis. After 2 h of incubation, cells were washed and incubated for an additional 60 min in RPMI-1640 + 100 µg/mL gentamicin. Macrophages were lysed with 0.1% saponin for 10 min at room temperature, and viable bacteria were enumerated as above. For PMN assays [39], 5 × 10^5^ cells were combined with 5 × 10^6^ CFUs of each isolate (MOI 10:1) in serum-free RPMI-1640 and synchronize by centrifugation at 524× *g* for 8 min at 4 °C. After 3 h, PMNs were lysed with 0.1% saponin, and viable bacteria were enumerated. Control groups of non-phagocyted bacteria were included.

### 2.5. Synthesis of the PSIR-3 Compound

The structural and photophysical characterization of the PSIR-3 compound was described previously [40]. The complex synthesized can be described by using the following general formula: [Ir(C^N)_2_(N^N)](PF_6_), where N^N is the ancillary ligand; and C^N corresponds to a cyclometalating ligand. In this study PSIR-3 is [Ir(ppy)_2_(ppdh)]PF_6_ where ppdh is pteridino(7,6-f)(1,10)phenanthroline-1,13(10H,12H)-dihydroxy and ppy is 2-phenylpyridine [41]. The structure and purities of the compound were confirmed by nuclear magnetic resonance (NMR), Fourier-transform infrared spectroscopy (FTIR), and mass spectroscopy (MALDI–MS) measurements. The absorption spectra were measured in acetonitrile (ACN) solutions using a Shimadzu UV–Vis Spectrophotometer UV-1900. The molar extinction coefficients of the characteristic bands were determined from the absorption spectra. Photoluminescence spectra were taken on an Edinburgh Instrument spectrofluorimeter using ACN solutions of the compounds previously degassed with N_2_ for approximately 20 min. The emission quantum yields (Φ_em_) were calculated according to the description of the literature [42]. Fluorescence lifetimes were measured by using a time-correlated single-photon counting (TC-SPC) apparatus (PicoQuant Picoharp 300) equipped with a sub-nanosecond LED source (excitation at 380 nm) powered by a PicoQuant PDL 800-B variable (2.5−40 MHz) pulsed power supply.

### 2.6. Antimicrobial Activity of Photosensitizer Compounds

Stock solutions of 2 g/L of the PSIR-3 compound solubilized in ACN were used to prepare working solutions in distilled water. For the antimicrobial assay, the collection of 118 clinical isolates of *K. pneumoniae* was used, and the control strains of susceptible *K. pneumoniae* KPPR1 and the MDR strain ST258 were also included. All bacteria were grown as axenic culture in Luria Bertani broth or agar medium as appropriate. PSIR-3 was mixed in 24-well plates at a final concentration of 4 mg/L for photodynamic experiments, with suspensions of 1 × 10^7^ colony forming units (CFUs)/mL of each bacterium, in a final volume of 500 µL of cation-adjusted Mueller-Hinton (ca-MH) broth. Exposure to light was performed for 30 min in a chamber with a white LED lamp at a photon flux of 17 µW/cm^2^. After exposure to light, the CFUs of the viable bacteria were determined by broth-micro dilution and sub-cultured on ca-MH agar plates. Following the recommendations of the Clinical and Laboratory Standards Institute (CLSI 2017) [34], the agar plates were incubated during 16–20 h at 37 °C, in the dark, and colony count was recorded, using a stereoscopic microscope. Control wells with bacteria culture with no photosensitizer or photosensitizer but not exposed to light were also included.

### 2.7. Determination of the Synergy between PSIR-3 and Cfx

The fractional inhibitory concentration index (FIC) value was determined using the following formula [43,44]. MICac is the MIC of a compound A, combined with a compound B, and MICbc is the MIC of the compound B combined with the compound A. The MICa and MICb are the MIC of the A and B compounds alone, respectively. Values in the FIC index ≤0.5 are considered synergistic, and values > 4 are considered antagonistic [44].

$$\text{FIC Index}=\frac{\text{MICac}}{{\text{MIC}}_{a}}+\frac{\text{MICbc}}{{\text{MIC}}_{b}}$$

To determine the MIC-Cfx combined with each PSs, 1 × 10^7^ UFC/mL of ESBL-producing bacteria were aPDI treated for 30 min with 4 mg/L of each PS and mixed with serial dilution (32–0.125 mg/L) of Cfx in ca-MH broth, as above. To determine the MIC-PSs combined with Cfx, 1 × 10^7^ UFC/mL of ESBL-producing bacteria was mixed with serial dilution of each PSs (32–0.125 mg/L) and a fixed concentration of 4 mg/L of Cfx, and then it was subjected to aPDI, as above.

### 2.8. Statistical Analyses

Statistical analyses were performed by using the Systat 13.2 software (Systat Software, Inc., San Jose, CA, USA) and GraphPad v6.01 (Prism) software. The X^2^ test or Fisher’s exact test for categorical variables and the Mann–Whitney *U* test for continuous non-parametric variables were used. The risk of virulence genes to modify the MIC of the antibiotic was established by determining the odds ratio with a CI: 95%.

## 3. Results

### 3.1. Demographic Characterization

**Figure 1:**
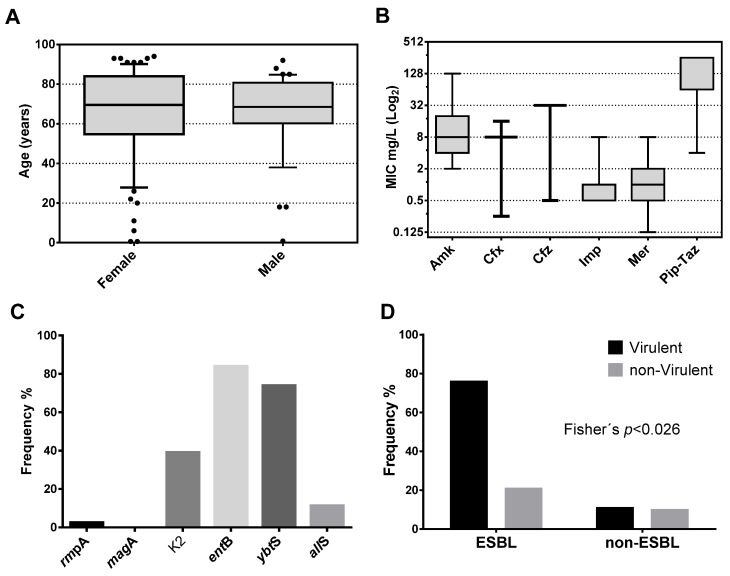
Characterization of patients and clinical isolates of *K. pneumoniae*. (**A**) Box plot for the age of patients stratified by gender. Patients show a median of 69.5 years for females and 68.5 years for males. (**B**) Box plot showing the median in the population of the minimum inhibitory concentration (MIC) of the seven antibiotics commonly used to treat infections by Gram-negative bacteria. (**C**) Frequency of carrying of the six genes evaluated in the study population. (**D**) Frequency distribution of carriers of three or more virulence factors stratified by extended-spectrum beta-lactamase (ESBL) production.

This work seeks to demonstrate the capacity of aPDI to inhibiting the growth of clinical isolates of *K. pneumoniae*, which are diverse in antimicrobial susceptibility, genotype, and virulence. Phenotypic and genotypic characterization was conducted to determine the frequency of genes encoding virulence factors, the MIC values determined for various antibiotics, and susceptibility to innate immune components. From the clinical isolates of *K. pneumoniae* received in the laboratory, 118 were selected from different unrelated patients. The isolates were recovered mainly from urine samples, 113 (95.8%), and only five from respiratory samples (three endotracheal aspirates (2.5%) and two expectorations (1.7%)). As shown in Table 2, the samples were obtained from 78 (66%) females and 40 (34%) males, from 12 clinical services, with a higher contribution from the emergency room 41 (34.75%), secondly the unit of medicine 24 (20.34%), third ambulatory 16 (13.56%) and in the fourth place urology 14 (11.86%). Of the 118 clinical isolates, 114 came from adults and 4 from pediatric patients. As shown in Figure 1A, the patient’s age fluctuated between 7 months and 94 years, with a median (IQR: 25–75%) of 69.5 (54.8–84) years for females, and between 10 months and 92 years, with a median of 68.5 (60.3–80.8) years for males. There are no significant differences in age between genders (*p* = 0.279 Mann–Whitney *U* test). The results confirm the infections with MDR and not MDR *K. pneumoniae* mainly affects the elderly population. The elderly are the most susceptible population to present complications derived from infectious diseases [45].

### 3.2. Antibiotic Susceptibility

In the *K. pneumoniae* population, the median and IQR (25–75%) of the MIC were determined and expressed in mg/L in a log_2_ box plot. As shown in Figure 1B, the bacterial population was mainly susceptible to Amk with a median (IQR) of 8 (4–20). Similar to Amk, the population was mainly susceptible to carbapenem, Imp, and Mer with a median of 1 (0.5–1) and 1 (0.5–2), respectively. In contrast, most of the population was resistant to the cephalosporins, Cfx, and Cfz, with medians of 8 (8–8) and 32 (32–32). Finally, similar to cephalosporin, almost the entire population was resistant to the Pip–Taz combination with a median of 256 (64–256). From the population, 66 isolates resistant to Cfx were susceptible to the combination Cfx/clavulanic acid. Those isolates were identified as ESBL-producing bacteria.

### 3.3. Virulence Gene Frequency

In this work, we select certain virulence genes belonging to families with different mechanisms of action. PCR determined the frequency of carrying genes encoding the virulence factors *rmp*A, *mag*A, K2, *ent*B, *all*S, and *ybt*S for each isolate using specific primers (Table 1). These genes were selected for being representatives of different families of virulence factors. As shown in Figure 1C, the harboring frequency was; *rmp*A [2.54% (3/118)], *mag*A [0% (0/118)], K2 [39% (46/118)], *ent*B [83.9% (99)], *all*S [11.2% 13/118)] and *ybt*S [73.8% (87/118)]. The most frequent virulence factor was the *ent*B gene, followed by the *ybt*S gene. There were no isolates with the *mag*A gene, and only three isolates harbor the *rmp*A gene. No isolates showed the hypermucoviscosity phenotype (by string test) in agar plates in the population of 118 unrelated isolates.

### 3.4. Correlation of Virulence Factors with Antibiotic Resistance

As shown in Figure 1D, there is a significantly higher frequency of ESBL strains that harbor three or more virulence factors compared to non-ESBL strains (Fisher’s; *p* < 0.026). The non-parametric Mann–Whitney *U* test (Systat 13 software) was used to determine the association that each virulence gene has on the median MIC value assuming a null hypothesis α = 5%. As shown in Table 3, the *rmp*A gene was not significantly associated with a modification of the median-MIC of any of the antibiotics analyzed in this study (*p* > 0.05). On the other hand, the *all*S gene significantly influenced the median-MIC of Cfx, Cfz, and Pip–Taz (*p* < 0.05). Similarly, the K2 gene significantly influenced the median-MIC of Cfx and Pip–Taz (*p* < 0.05). The *ent*B gene significantly affected the median-MIC of Amk and Imp (*p* < 0.05). The *ybt*S gene significantly influenced the median-MIC of Cfx (*p* < 0.006). Finally, the *all*S gene significantly influenced the median-MIC of Cfx and Cfz antibiotics (*p* < 0.05). The MIC of the antibiotics more sensitive to the presence of virulence factors were the Cfx (sensitive to K2, *ybt*S, and *all*S genes) and Pip–Taz (sensitive to the K2 and *all*S genes). These data show that, regardless of the patient’s conditions, the multi-virulence is effectively an independent risk factor that promotes ESBL-production of clinical populations of *K. pneumoniae* with a *p* < 0.026 Fisher’s exact test.

**Figure 2:**
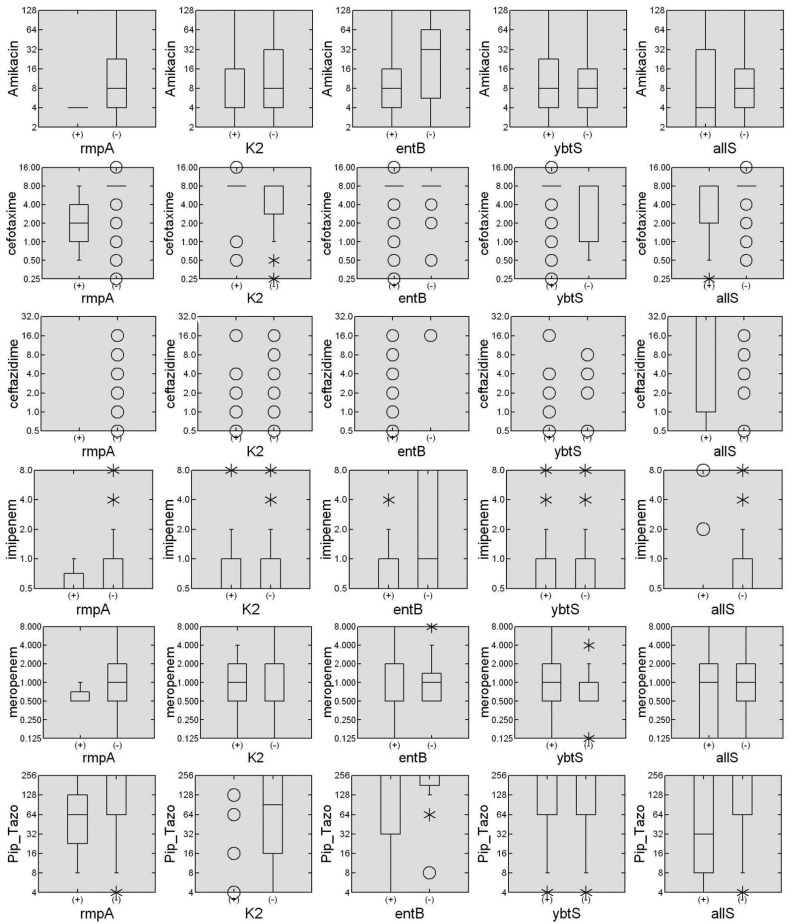
Association of virulence genes in the measurement of median MIC. The box diagrams created with the Systat 13 software summarize the MIC measures for six commonly used antibiotics, stratified by the presence or absence of each of the six virulence genes. The data are presented as mg/L in a Log_2_ scale of MICs for each antibiotic stratified by the presence (+) or absence (−) of each virulence gene. The horizontal lines represent the median and the frequency limits between 25 and 75% of the individuals, the hollow dots represent single individuals, and the asterisks represent small groups of individuals.

As shown in Figure 2, the box plot for each antibiotic stratified by the presence or absence of virulence genes was constructed to verify if the influence is to increase or decrease the median of the MIC. The presence of the *ent*B gene is associated with a decrease in the median-MIC for Amk and Imp. On the other hand, the K2 gene is associated with an increase in the median-MIC for Cfx and Pip–Taz. Similarly, the *ybt*S gene is associated with an increase in the median-MIC for Cfx. Finally, the *all*S gene is associated with an increased median-MIC for Cfz and decreased median-MIC for Cfx and Pip–Taz. Some of the virulence factors studied here were associated with the median- MIC modification for several antibiotics. In the *K. pneumoniae* population tested, the most influencing virulence factors were *ent*B, *ybt*S, and *all*S genes. The stratified MICs-box plot made it possible to distinguish how virulence genes modulate antibiotic susceptibility by increasing or decreasing. Remarkably, the sum of the genes of the K2^+^, *ybt*S^+^, and *all*S^+^ genes contributes to increasing the median-MICs to values higher than the clinical susceptibility breakpoint established by the CLSI. These increased values occur similarly when efflux pumps are activated in other Gram-negative bacteria [46]. For example, in a population of *Pseudomonas aeruginosa*, the combined overexpression of the *mex*A and *mex*X efflux pump increased the median MIC for ciprofloxacin and cefepime above the cutoff points [46,47].

### 3.5. Association of K2^+^, ybtS^+^, and allS^+^ Virulence Genes to Survive the Innate Immunity

Our results show that the K2 and *ybt*S virulence genes are risk factors for the production of ESBL and that the *all*S gene acts as a protective factor. Then, we selected two groups of clinical isolates of *K. pneumoniae*, which harbor or not these three genes, to assess their susceptibility to innate immunity components. Only three isolates, identified by the numbers 81, 92, and 111, are of the K2^+^, *ybt*S^+^, and *all*S^+^ genotype, and unexpectedly all of them are ESBL-producers. From the genotype K2^−^, *ybt*S^−^ and *all*S^−^, the isolates 13, 21, and 22 were selected as being non-ESBL. As controls, the susceptible but highly virulent *K. pneumoniae* KPPR1 (K2^+^, *ybt*S^+^, and *all*S^+^) and the MDR but less virulent ST258 (K2^−^, *ybt*S^−^ and *all*S^−^) strains were also included.

**Figure 3:**
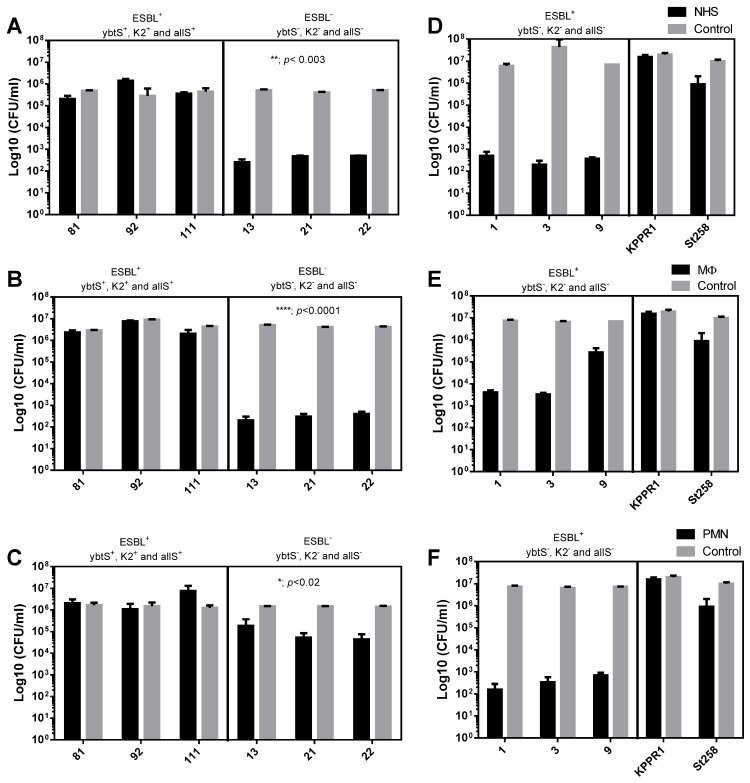
Effect of virulence genes on the susceptibility of ESBL or non-ESBL strains to components of innate immunity. The susceptibility of ESBL-producing *K. pneumoniae* clinical isolates bearing the virulence genes *ybt*S^+^, K2^+^, and *all*S^+^ was determined and compared with non-ESBL producing bacteria that lack the virulence genes. (**A**,**D**) Susceptibility to normal human serum (NHS), (**B**,**E**) susceptibility to phagocytosis by macrophages (MΦ), and (**C**,**F**) susceptibility to phagocytosis by polymorphonuclear cells (PMN). The results of two independent experiments performed in triplicate are shown (n = 6). Viable bacteria were enumerated by colony count on ca-MH agar after serial micro-dilution. The colony forming units (CFUs)/mL values are presented as means +/− SD, on a log_10_ scale of treated bacteria (black bars) compared to untreated control bacteria (gray bars). **** = *p* < 0.0001, ** = *p* < 0.003 and * = *p* < 0.02 of two-way ANOVA comparing the proportion of treated/untreated ESBL bacteria with non-ESBL bacteria.

To determine the bacterial susceptibility to normal human serum (NHS), they were exposed for 3 h, and then the number of viable bacteria was determined by microdilution and plate counting. As shown in Figure 3A, the presence of all three virulence genes, K2^+^, *ybt*S^+^, and *all*S^+^, significantly (** = *p* < 0.003; two-way ANOVA) increases the survival of ESBL-producing bacteria compared to non-ESBL bacteria lacking all three virulence genes. On average, the serum resistance was improved by four orders of magnitude (4log_10_). Similar to that observed with serum, ESBL-producing bacteria of the K2^+^, *ybt*S^+^, and *all*S^+^ genotype show a significant (**** = *p* < 0.0001) increase, 4log_10_, in the survival to the MΦ activity, compared to no- ESBL bacteria that lacks these virulence genes (Figure 3B). Finally, comparable to MΦ, survival to the activity of PMNs of the bacteria of genotype K2^+^, *yb*tS^+^, and *all*S^+^ increased significantly (* = *p* < 0.02), at least one time on average, compared to non-ESBL strains that lack the virulence genes (Figure 3C). As shown in Figure 3, the virulent control KPPR1 strain was more resistant to serum (Figure 3D), macrophages (Figure 3E), and PMN (Figure 3F) compared to the MDR ST258 *K. pneumoniae* strain. Consistently the clinical control isolates have shown to be more susceptible to the bactericidal activity of the serum (3D) and the phagocytic activity of MΦ (3E) and PMN (3F). The co-occurrence of harboring multiple genes that encode virulence factors and the ESBL-production leads to enhanced virulence. The ESBL-producing strains of *K. pneumoniae* of the genotype K2^+^, *ybt*S^+^, and *all*S^+^ were more resistant to innate immunity, consistently with studies over MDR populations of *K. pneumoniae* that increased their 30-day mortality over patients undergoing bloodstream infections [1,48]. Moreover, virulence genes, such as siderophores, which have an essential role in bacterial survival and virulence [49,50], have been previously associated with the MDR-*K. pneumoniae* [16,51]. In this study, ESBL-producer *K. pneumoniae* of the K2^+^, *ybt*S^+^, and *all*S^+^ genotype shown a survival improvement for killing by PMNs. Previously, the PMN has demonstrated a limited binding and uptake capacity for MDR–*K. pneumoniae* [39]. The activity of the PMN is the most important cellular component of the innate immune response, essential as the first line of defense against bacterial infections [52].

### 3.6. Susceptibility of Clinical Isolates to aPDI with PSIR-3

#### 3.6.1. Photophysical Properties of the PSIR-3 Compound

**Figure 4:**
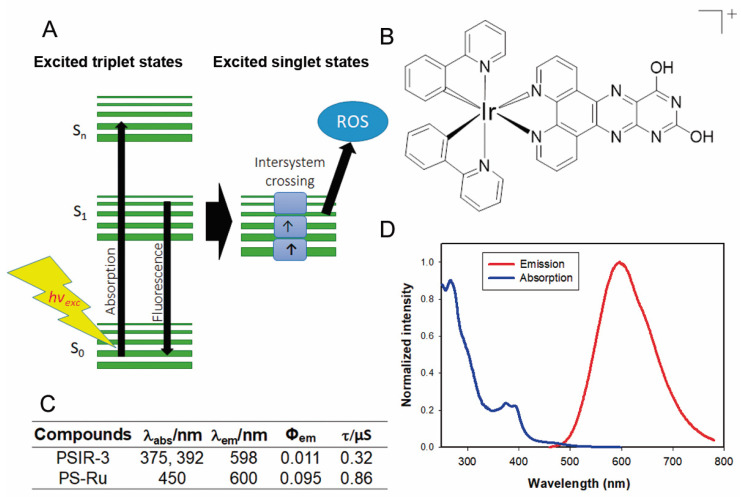
The photophysical properties of the PSIR-3 photosensitizer. (**A**) schematic representation of the absorption–emission process of photosensitizer molecules. Light excites external electrons to accede from a basal state S_0_ to a higher energetic state S_1_–S_n_. Suppose the electrons return to its ground state; the energy is then released as fluorescence. However, when the excited electron enters an intersystem crossing process, the released energy excites molecular oxygen and converts it to reactive oxygen species (ROS). (**B**) chemical structure of the Ir(III) compound (PSIR-3, [Ir(ppy)_2_(ppdh)]PF_6_). (**C**) Summary of the photophysical properties of the PSIR-3 and PS-Ru compounds, where λ_abs_ = wavelength of absorbance, λ_em_ = wavelength of emission, Φ_em_ = emission quantum yield, and τ = time in the excited state_._ Data for PS-Ru were obtained from the literature. Adapted from [42], Elsevier, 2010; Adapted from [53], American Chemical Society, 1983. (**D**) The absorption and the emission spectra of the PSIR-3 in acetonitrile (ACN).

We have previously shown that Ir(III)-based compounds, such as PSIR-3, have photodynamic antimicrobial activity against imipenem-resistant *Klebsiella pneumoniae* [32,33]. In this work, we tested a coordination compound characterized by a positive charge in the first coordination sphere (Figure 4B). The photophysical evaluation of the PSIR-3 performed in acetonitrile solution [40] revealed absorption processes at 375 and 392 nm attributable at the first instance of charge-transfer transitions (Figure 4C,D) [32]. When the PSIR-3 compound was excited with a wavelength corresponding to the lowest charge-transfer absorption energy, 375 nm, it showed maximum emission at 598 nm (Figure 4C,D). Figure 4C shows the recorded lifetimes of excited states in 0.32 μs and the calculated quantum yield (Φ_em_) in 0.011 [40]. The aPDI activity of the PSIR-3 compound was compared with the positive PS control [Ru(bpy)_3_](PF_6_)_2_ (bpy = 2,2′-bipyridine) called PS-Ru. According to the literature, the PS-Ru shows a charge-transfer absorption process at 450 nm with maximum emission at 600 nm (excited in 450 nm) in acetonitrile [42], with Φ_em_ of 0.095 [42], and a lifetime registered of its excited state of 0.855 μs (Figure 4C) [53]. The maximum absorption of PSIR-3 occurs at wavelengths below 400 nm, that although it is more energetic, it penetrates the tissues poorly. Therefore, this compound will activate better if it is directly irradiated, such as in superficial wounds. However, UTI is one of the most common diseases caused by *K. pneumoniae*, where probes that deliver the light dose within internal surfaces can irradiate the epithelial lining of the bladder [54]. Certain kinds of fiber arrays or inflatable balloons may provide a homogenous light power delivery [55,56]. This catheterization can be applied for inpatients suffering UTI that does not respond to antibiotic treatment.

#### 3.6.2. Antimicrobial Photodynamic Inhibition of the PSIR-3 over Clinical Isolates

**Figure 5:**
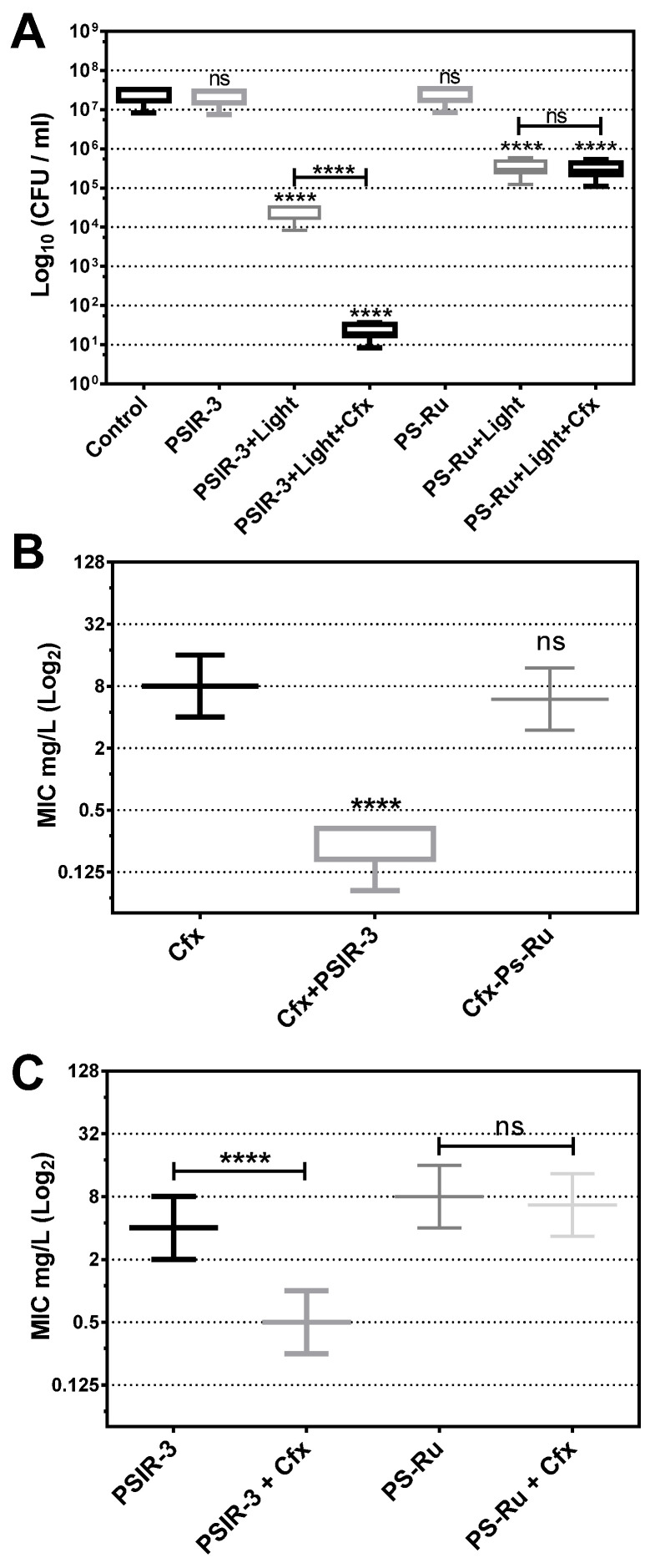
Antimicrobial photodynamic inactivation of clinical isolates of *K. pneumoniae*. (**A**) Growth inhibition of 118 clinical isolates of *K. pneumoniae* subjected to antimicrobial photodynamic inactivation (aPDI) with PSIR-3. The bacteria were used at a concentration of 1 × 10^7^ CFUs/mL and mixed in triplicate with 4 mg/L of PSIR-3 or PSIR-4 compounds. For the aPDI, the mixture of bacteria with PS was exposed for 1 h at 17 µW/cm^2^ of white light. As a control, bacteria combined with the photosensitizers (PSs) not exposed to light (PSIR-3 or PS-Ru) and bacteria not combined with the PSs (control) were included. Colony count enumerated of viable bacteria on ca-MH agar after serial micro-dilution. The CFUs/mL values are presented as means ± SD on a log_10_ scale. (**B**) From the clinical isolates, 66 ESBL-producing bacteria were exposed to aPDI, using PSIR-3 or PS-Ru, and MIC for cefotaxime (Cfx) was performed in triplicate, in ca-MH broth, for 16–20 h. (**C**) For ESBL-producing bacteria, the MIC for PSIR-3 or PS-Ru, were determined in combination with 4 mg/L of Cfx, performed in triplicate in ca-MH agar for 16–20 h. The MIC values are presented as median ± SD of mg/mL on a log_2_ scale. Not significant (ns) *p* > 0.05 by Student’s *t*-test among treated bacteria compared to control; **** *p* < 0.0001 by Student’s *t*-test among treated bacteria compared to control.

Photodynamic treatment was verified to produce the observed growth inhibition of the 118 clinical isolates of *K. pneumoniae* compared to the untreated bacteria. The photodynamic activity of the PSIR-3 compound was compared to the activity of the PS-Ru reference compound as a positive control [41,57,58,59]. As seen in Figure 5, compared to the control of untreated bacteria, photodynamic treatment with 4 µg/mL PSIR-3 inhibits bacterial growth > 3 log_10_ (>99.9%) of clinical isolates of *K. pneumoniae* (**** *p* < 0.0001; compared to untreated control). The results show that the bactericidal effect produced by PSIR-3 is light-dependent (ns = *p* > 0.05; compared to the untreated control). These results are comparable with those obtained with the positive control compound PS-Ru, which has shown that bacterial growth inhibition is light-dependent (**** *p* < 0.0001; compared to the untreated control).

#### 3.6.3. Synergism between aPDI with PSIR-3 and Cefotaxime

Since PSIR-3 showed synergism combined with imipenem [32], we analyzed whether it shows synergism with cefotaxime in the population of clinical isolates. First, it was determined whether the combined treatment with Cfx increases the inhibition of bacterial growth of aPDI with PSIR-3. The 118 clinical isolates of *K. pneumoniae* were exposed to the preparation of 4 µg/mL of cefotaxime with 4 µg/mL of PSIR-3 (its MIC). Control bacteria without Cfx and exposure to light were included. As expected, the PSIR-3 compound mixed with cefotaxime significantly (**** *p* < 0.0001) increased the bactericidal effect on the clinical isolates population from 3 to 6 log_10_ reduction (Figure 5A). As seen before, a significantly increased inhibitory effect was not observed when combining the cefotaxime with the PS-Ru control compound (ns *p* > 0.05). Secondly, the set of 66 clinical isolates characterized as ESBL-producing *K. pneumoniae* were treated with PSIR-3 aPDIfor 1 h, and serial dilutions determined the MIC for Cfx in ca-MH broth. As seen in Figure 5B, a significant reduction (**** *p* < 0.0001) from 8 µg/mL (8–8) to 0.17 µg/mL (0.17–0.333) on Cfx-MIC was observed compared to the untreated group. The combined treatment also reduced the PSIR-3-MIC, from 4 to 0.5 mg/L (Figure 5C). Similar to previously shown with imipenem [32], compound PSIR-3 produced a significant change in Cfx-susceptibility with a fractional inhibitory concentration (FIC) index of 0.15 (Table 4). Figure 5C shows the control compound, PS-Ru, did not significantly change Cfx-susceptibility with an FIC Index 1.58 (Table 4). Because synergy is defined as an FIC index of ≤0.5 [44], the increased inhibitory effect observed when combining PSIR-3 with Cfx is not summative but synergistic.

The behavior exhibited by PSIR-3 must be related, as mentioned in previous reports [32,33], to the external substituents bonded to polypyridine ligand structures and affinity to bacterial envelope [41]. This synergism is also comparable to other photosensitizers; for example, rose bengal showed an increase in the susceptibility of *Acinetobacter baumannii* for a range of antibiotics used along with aPDI [60]. In another example, conventional antibiotics and alternative compounds reported synergism in a murine model for pathogens of the ESKAPE group (*Enterococcus faecium*, *Staphylococcus aureus*, *Klebsiella pneumoniae*, *Acinetobacter baumannii*, *Pseudomonas aeruginosa*, and *Enterobacter* species) [61], using anti-biofilm peptides [62]. For now, it is difficult to accurately calculate the dose of light necessary to activate PSIR-3 effectively; however, we observed in vitro that with low doses of energy (17 µW/cm^2^ of white light), it is bactericidal. Compounds with optimum absorbance at higher wavelengths, bordering the 630–750 nm, would improve the exposure of PS to light into the tissues, but being less energetic, the PSs must have a triplet excited state of easy access to promote energy transfer [63]. We, therefore, need to perform a better characterization of its antimicrobial activity when activated with defined wavelengths, before starting in vivo studies, in urinary infection models and to evaluate the need to deliver light through intraurethral catheters [64].

## 4. Conclusions

In this work, we saw that multi-drug resistance and virulence are significant factors in clinical isolates of *K. pneumoniae*. However, the increase in MICs can be neutralized by aPDI, turning resistant strains susceptible. APDI is effective in treating multidrug-resistant bacteria and more virulent strains, as well as strains that combine both characteristics. The aPDI then becomes a great support to antimicrobial therapy in a shortage of new effective antibiotics. The photophysical characterization of PS indicates that its maximum absorption occurs at wavelengths lower than 400 nm, which could constitute a problem for its use in infections of internal organs due to low penetration. In UTI, optical fibers can be used through a catheter to deliver the dose of light [54,55]. Moreover, compounds with optimum absorbance at higher wavelengths, bordering the 630–750 nm, would improve the exposure of PS to light in the tissues.

## Figures and Tables

**Table 1:** Primers for genes encoding virulence factors of *Klebsiella pneumoniae.*

Gene	Primers	Gene Type	Amplicon Size
*ybt*S	GACGGAAACAGCACGGTAAA	Siderophores	242
	GAGCATAATAAGGCGAAAGA		
*ent*B	GTCAACTGGGCCTTTGAGCCGTC	Siderophores	400
	TATGGGCGTAAACGCCGGTGAT		
*mag*A	GGTGCTCTTTACATCATTGC	Capsular serotype K1 and hypermucoviscosity phenotype	128
	GCAATGGCCATTTGCGTTAG		
*rmp*A	CATAAGAGTATTGGTTGACAG	Regulator of mucoid phenotype A	461
	CTTGCATGAGCCATCTTTCA		
K2	CAACCATGGTGGTCGATTAG	Capsular serotype K2 and hypermucoviscosity phenotype	531
	TGGTAGCCATATCCCTTTGG		
*all*S	CATTACGCACCTTTGTCAGC	Allantoin metabolism	764
	GAATGTGTCGGCGATCAGCTT		
16S	ATTTGAAGAGGTTGCAAACGAT	Gene encoding the 16S ribosomal RNA	133
	TTCACTCTGAAGTTTTCTTGTGTTC		

**Table 2:** Frequency of *K. pneumoniae* isolation by hospital unit and genre.

Service	Female	Male	Total	Percent
Outpatient	12	4	16	13.56%
surgery	5	5	10	8.47%
Endocrinology	1	0	1	0.85%
Geriatrics	1	2	3	2.54%
Gynecology	2	0	2	1.69%
Home hospitalization	1	0	1	0.85%
Medicine	2	0	24	20.34%
Medical–surgical service	13	11	2	1.69%
Pediatrics	1	0	1	0.85%
Critical patient unit	2	1	3	2.54%
Emergency room	31	10	41	34.75%
Urology	7	7	14	11.86%
TOTAL	78	40	118	100%

**Table 3:** Mann–Whitney *U* test *p*-values for modification of antibiotic MIC by virulence genes.

	Amikacin	Cefotaxime	Ceftazidime	Imipenem	Meropenem	Pip–Taz
*rmp*A	0.285	0.051	0.432	0.363	0.527	0.411
K2	0.158	**0.002**	0.919	0.102	0.588	**0.001**
*ent*B	**0.009**	0.918	0.117	**0.015**	0.291	0.072
*ybt*S	0.572	**0.006**	0.722	0.340	0.502	0.067
*all*S	0.326	**0.024**	**0.001**	0.086	0.537	**0.013**

Values in bold represent a *p* < 0.05, below the null hypothesis value with α = 5%, which means a significant difference in MIC due to the presence of the virulence factor. Pip–Taz, piperacillin–tazobactam.

**Table 4:** Fractional inhibitory concentration (FIC) index calculation.

Compounds	MIC	MIC Combined	FIC	FIC Index
Cfx	8.00			
PSIR-3	4.00	0.17	0.02	0.15
PSIR-3 *		0.50	0.13	
PS-Ru	8.00	6.67	0.83	1.58
PS-Ru *		6.00	0.75	

MIC values are de median for the ESBL-producing *K. pneumoniae*, n = 66. * The MIC value modified by Cfx.

## Data Availability

The data presented in this study are available on request from the corresponding author. The data are not publicly available because they are confidential data of patients protected by the informed consent protocol.
